# Supplementary material for: Comprehensive Annotation and Functional Exploration of MicroRNAs in Lettuce
Source: Front Plant Sci. 2021 Dec 24;12:781836. doi: 10.3389/fpls.2021.781836 (PMC8739914; doi:10.3389/fpls.2021.781836)
Supplement: Supplementary file 2 [file Data_Sheet_8.ZIP › GSM2230747.plot/Lsa-miR394c_Lsat_1_v5_gn_6_102440.1_1294_TPlot.pdf]

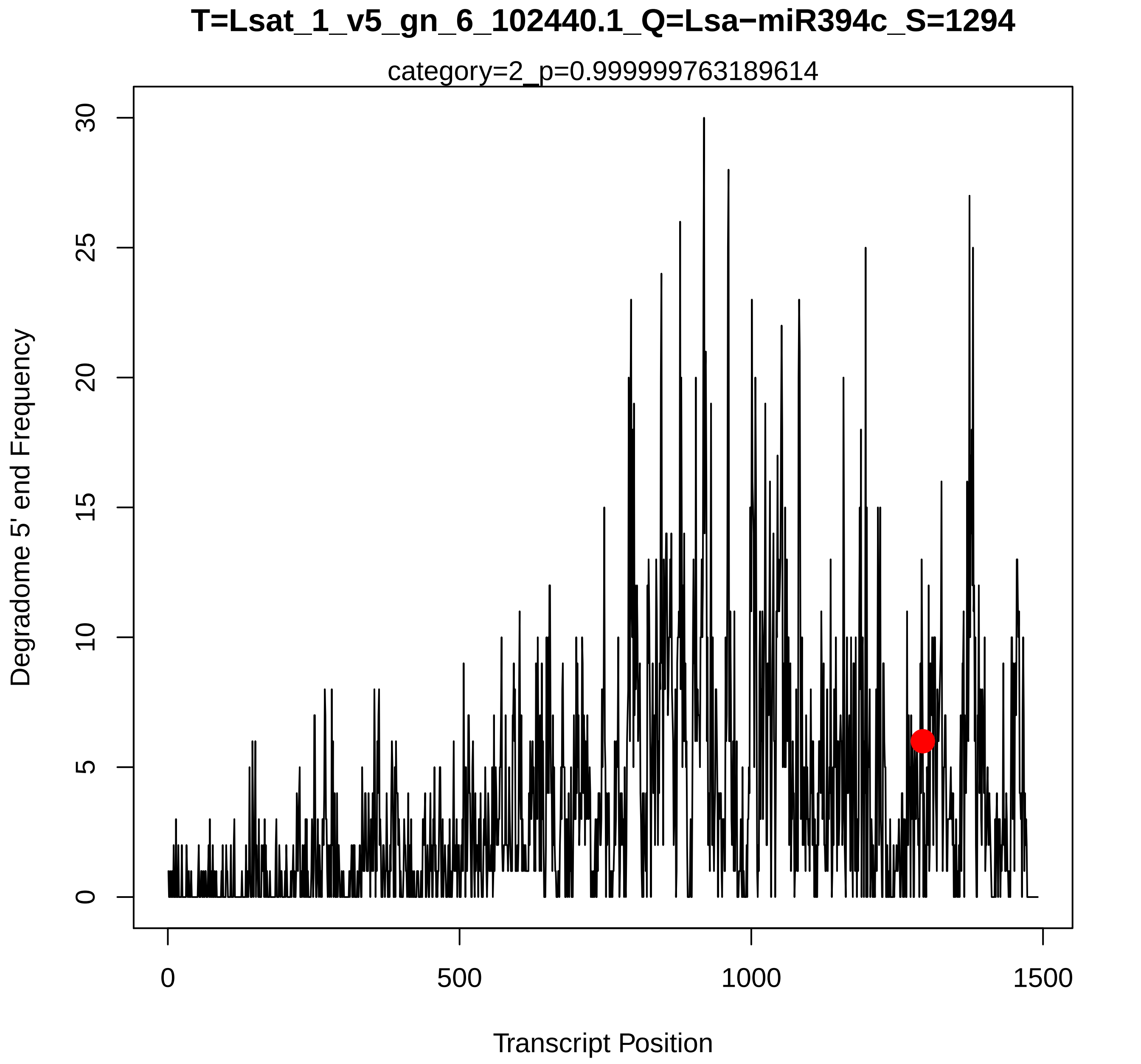
T=Lsat_1_v5_gn_6_102440.1_Q=Lsa−miR394c_S=1294
category=2_p=0.999999763189614
Degradome 5' end Frequency
Transcript Position
0
5
10
15
20
25
30
0
500
1000
1500
